# Periodic Solutions for Nonlinear Integro-Differential Systems with Piecewise Constant Argument

**DOI:** 10.1155/2014/514854

**Published:** 2014-01-12

**Authors:** Kuo-Shou Chiu

**Affiliations:** Departamento de Matemática, Facultad de Ciencias Básicas, Universidad Metropolitana de Ciencias de la Educación, Santiago, Chile

## Abstract

We investigate the existence of the periodic solutions of a nonlinear integro-differential system with piecewise alternately advanced and retarded argument of generalized type, in short DEPCAG; that is, the argument is a general step function. We consider the critical case, when associated linear homogeneous system admits nontrivial periodic solutions. Criteria of existence of periodic solutions of such equations are obtained. In the process we use Green's function for periodic solutions and convert the given DEPCAG into an equivalent integral equation. Then we construct appropriate mappings and employ Krasnoselskii's fixed point theorem to show the existence of a periodic solution of this type of nonlinear differential equations. We also use the contraction mapping principle to show the existence of a unique periodic solution. Appropriate examples are given to show the feasibility of our results.

## 1. Introduction

Among the functional differential equations, Myshkis [1] proposed to study differential equations with piecewise constant arguments: DEPCA. The theory of scalar DEPCA of the type

(1) dx(t)dt=f(t,x(t),x(γ(t))),γ(t)=[t] or γ(t)=2[t+12],

where [·] signifies the greatest integer function, was initiated in [2–4], in Wiener [5], the first book in DEPCA, and has been developed by many authors [6–21]. Applications of DEPCA are discussed in [5, 22–25]. They are hybrid equations; they combine the properties of both continuous systems and discrete equations. Over the years, great attention has been paid to the study of the existence of periodic solutions of several different types of differential equations. For specific references see [5–7, 13, 17, 18, 23, 24, 26–34].

Let *ℤ*, *ℕ*, ℝ, and *ℂ* be the set of all integers, natural, real, and complex numbers, respectively. Denote by |·| a norm in ℝ^*n*^, *n* ∈ *ℕ*. Fix two real sequences *t*_*i*_, *γ*_*i*_, *i* ∈ *ℤ*, such that *t*_*i*_ < *t*_*i*+1_,  and *t*_*i*_ ≤ *γ*_*i*_ ≤ *t*_*i*+1_ for all *i* ∈ *ℤ*, and  *t*_*i*_ → ±*∞* as *i* → ±*∞*.

Let *γ* : ℝ → ℝ be a step function given by *γ*(*t*) = *γ*_*i*_ for *t* ∈ *I*_*i*_ = [*t*_*i*_, *t*_*i*+1_) and consider the DEPCA (1) with this general *γ*. In this case we speak of DEPCA of general type, in short DEPCAG. Indeed, *γ*(*t*) = [*t*] corresponds to *γ*_*i*_ = *t*_*i*_ = *i* ∈ *ℤ*, and *γ*(*t*) = 2[(*t* + 1)/2] corresponds to *t*_*i*_ = 2*i* − 1, *γ*_*i*_ = 2*i*, *i* ∈ *ℤ*. The particular case of DEPCAG, when *γ*_*i*_ = *t*_*i*_, *i* ∈ *ℤ*, an only delayed situation, is considered by first time in Akhmet [8]. The other extreme case is the only advanced situation *γ*_*i*_ = *t*_*i*+1_. Any other situation means an alternately advanced and delayed situation with *I*_*i*_^+^ = [*t*_*i*_, *γ*_*i*_] the advanced intervals and *I*_*i*_^−^ = [*γ*_*i*_, *t*_*i*+1_) the delayed intervals. In [15, 16], Pinto has cleared the importance of the advanced and delayed intervals. This decomposition will be present in all our results. See [12, 13, 15, 16, 23, 24, 35]. The integration or solution of a DEPCA, as proposed by its founders [2–4, 6], is based on the reduction of DEPCA to discrete equations. To study nonlinear DEPCAG, we will use the approach proposed by Akhmet in [9], based on the construction of an equivalent integral equation, but we also remark the clear influence of the discrete part and the corresponding difference equations will be fundamental.

In 2008, Akhmet et al. [10] obtained some sufficient conditions for the existence and uniqueness of periodic solutions for the following system:

(2) $$\begin{array}{c} x^{\prime }\left(t\right)=A\left(t\right)x\left(t\right)+h\left(t\right)+\mu g\left(t,x\left(t\right),x\left(\gamma \left(t\right)\right),\mu \right), \end{array}$$

where *A* : ℝ → ℝ^*n*×*n*^, *h* : ℝ → ℝ, and  *g* : ℝ × ℝ^*n*^ × ℝ^*n*^ × *I* → ℝ^*n*^ are continuous functions, *γ*(*t*) = *t*_*i*_ if *t*_*i*_ ≤ *t* < *t*_*i*+1_, and *μ* is a small parameter belonging to an interval *I* ⊂ ℝ with 0 ∈ *I*.

Recently, Chiu and Pinto [23], using Poincaré operator, a new Gronwall type lemma and fixed point theory, obtained some sufficient conditions for the existence and uniqueness of periodic (or harmonic) and subharmonic solutions of quasilinear differential equation with a general piecewise constant argument of the form

(3) $$\begin{array}{c} y^{\prime }\left(t\right)=A\left(t\right)y\left(t\right)+f\left(t,y\left(t\right),y\left(\gamma \left(t\right)\right)\right), \end{array}$$

where *t* ∈ ℝ, *y* ∈ *ℂ* ^*p*^, *A*(*t*) is a *p* × *p* matrix for *p* ∈ *ℕ*, *f*(*t*, *x*, *y*) is a *p* dimensional vector and *f* is continuous in the first argument, and *γ*(*t*) = *γ*_*i*_, if *t*_*i*_ ≤ *t* < *t*_*i*+1_, *i* ∈ *ℤ*. In this paper, comparing the three DEPCAG inequalities of Gronwall type and remarked new Gronwall lemma not only requests a weaker condition than the other Gronwall lemmas but also has a better estimate.

It is well-known that there are many subjects in physics and technology using mathematical methods that depend on the linear and nonlinear integro-differential equations, and it became clear that the existence of the periodic solutions and its algorithm structure from more important problems in the present time. Where many of studies and researches [36–40] dedicates for treatment the autonomous and non-autonomous periodic systems and specially with the integral equations and differential equations and the linear and nonlinear differential and which is dealing in general shape with the problems about periodic solutions theory and the modern methods in its quality treatment for the periodic differential equations.

Samoilenko and Ronto [41] assume the numerical-analytic method to study the periodic solutions for ordinary differential equations and its algorithm structure and this method includes uniform sequences of periodic functions and the results of that study are using the periodic solutions on wide range in the difference of new processes industry and technology. For example, Samoilenko and Ronto [41] investigated the existence and approximation of periodic solution for nonlinear system of integro-differential equations which has the form

(4) $$\begin{array}{c} x^{\prime }\left(t\right)=f\left(t,x\left(t\right),\overset{t+T}{\underset{t}{\int }}g\left(s,x\left(s\right)\right)ds\right), \end{array}$$

where *x* ∈ *D* ⊂ ℝ^*n*^; *D* is a closed and bounded domain. The vectors functions *f*(*t*, *x*, *y*) and *g*(*t*, *x*) are continuous functions in *t*, *x*, *y* and periodic in *t* of period *T*.

Butris [42] investigated the periodic solution of nonlinear system of integro-differential equations depending on the gamma distribution, by using the numerical analytic method, which has the form

(5) x′(t)=f(t,β(t,α),x(t),∫tt+Tg(s,β(s,α),x(s))ds),t∈ℝ,

where *x* ∈ *D* ⊂ ℝ^*n*^; *D* is a closed and bounded domain. The vector functions *f*(*t*, *β*(*t*, *α*), *x*) and *g*(*t*, *β*(*t*, *α*), *x*) are defined on the domain (*t*, *β*(*t*, *α*), *x*) ∈ ℝ × [0, *T*] × *D* × *D*_1_.

In the current paper, we study the existence of periodic solutions of a nonlinear integro-differential system with piecewise alternately advanced and retarded argument:

(6) z′(t)=A(t)z(t)+f(t,z(t),∫tt+ωC(t,s,z(γ(s)))ds) +g(t,z(t),z(γ(t))), t∈ℝ,

where *A* : ℝ → ℝ^*n*×*n*^, *f* : ℝ × ℝ^*n*^ × ℝ^*n*^ → ℝ^*n*^, and *g* : ℝ × ℝ^*n*^ × ℝ^*n*^ → ℝ^*n*^ are continuous in their respective arguments. In the analysis we use the idea of Green's function for periodic solutions and convert the nonlinear integro-differential systems with DEPCAG (6) into an equivalent integral equation. Then we employ Krasnoselskii's fixed point theorem and show the existence of a periodic solution of the nonlinear integro-differential systems with DEPCAG (6) in Theorem 12. We also obtain the existence of a unique periodic solution in Theorem 14 employing the contraction mapping principle as the basic mathematical tool. Furthermore, appropriate examples are given to show the feasibility of our results.

In our paper we assume that the solutions of the nonlinear integro-differential systems with DEPCAG (6) are continuous functions. But the deviating argument *γ*(*t*) is discontinuous. Thus, in general, the right-hand side of the DEPCAG system (6) has discontinuities at moments *t*_*i*_ ∈ ℝ, *i* ∈ *ℤ*. As a result, we consider the solutions of the DEPCAG as functions, which are continuous and continuously differentiable within intervals [*t*_*i*_, *t*_*i*+1_), *i* ∈ *ℤ*. In other words, by a solution *z*(*t*) of the DEPCAG system (6) we mean a continuous function on ℝ such that the derivative *z*′(*t*) exists at each point *t* ∈ ℝ, with the possible exception of the points *t*_*i*_ ∈ ℝ, *i* ∈ *ℤ*, where a one-sided derivative exists, and the nonlinear integro-differential systems with DEPCAG (6) are satisfied by *z*(*t*) on each interval (*t*_*i*_, *t*_*i*+1_), *i* ∈ *ℤ* as well.

The rest of the paper is organized as follows. In Section 2, some definitions and preliminary results are introduced. We show double *ω*-periodicity of Green's function.  Section 3 is devoted to establishing some criteria for the existence and uniqueness of periodic solutions of the DEPCAG system (6). Green's function and Banach, Schauder, and Krasnoselskii's fixed point theorems below are fundamental to obtain the main results. Furthermore, appropriate examples are provided in Section 4 to show the feasibility of our results.

## 2. Green's Function and Periodicity

In this section we state and define Green's function for periodic solutions of the nonlinear integro-differential system with piecewise alternately advanced and retarded argument (6).

Let *I* be the *n* × *n* identity matrix. Denote by Φ(*t*, *s*), Φ(*s*, *s*) = *I*, *t*, *s* ∈ ℝ, the fundamental matrix of solutions of the homogeneous system (7).

For every *t* ∈ ℝ, let *i* = *i*(*t*) ∈ *ℤ* be the unique integer such that *t* ∈ *I*_*i*_ = [*t*_*i*_, *t*_*i*+1_).

From now on the following assumption will be needed.

(*N*_*ω*_) The homogenous equation
  (7) $$\begin{array}{c} y^{\prime }\left(t\right)=A\left(t\right)y\left(t\right) \end{array}$$

does not admit any nontrivial *ω*-periodic solution.

**Remark 1**

For *τ* ∈ ℝ, the condition (*N*_*ω*_) equivalent to the matrix (*I* − Φ(*τ* + *ω*, *τ*)) is nonsingular.

Now, we solve the DEPCAG system (6) on *I*_*i*(*τ*)_ = [*t*_*i*(*τ*)_, *t*_*i*(*τ*)+1_):

(8) z′(t)=A(t)z(t)+f(t,z(t),∫tt+ωC(t,s,z(γ(s)))ds) +g(t,z(t),z(γi(τ))), t∈[ti(τ),ti(τ)+1),

which has the solution given by

(9) z(t)=Φ(t,τ)z(τ) +∫τtΦ(t,s)[f(s,z(s),∫sti(τ)+1C(s,u,z(γi(τ)))du+∑k=i(τ)+1i(s+ω)−1∫tktk+1C(s,u,z(γk))du+∫ti(s+ω)s+ωC(s,u,z(γi(s+ω)))du)+g(s,z(s),z(γi(τ)))]ds.

For *t* → *t*_*i*(*τ*)+1_ in (9), we have

(10) z(ti(τ)+1)=Φ(ti(τ)+1,τ)z(τ) +∫τti(τ)+1Φ(ti(τ)+1,s)×[f(s,z(s),∫sti(τ)+1C(s,u,z(γi(τ)))du+∑k=i(τ)+1i(s+ω)−1∫tktk+1C(s,u,z(γk))du+∫ti(s+ω)s+ωC(s,u,z(γi(s+ω)))du)+g(s,z(s),z(γi(τ)))]ds

and in general, by induction, for any *i*(*t*) ≥ *i*(*τ*),

(11) z(t)=Φ(t,τ)z(τ) +∫τti(τ)+1Φ(t,s)×[f(s,z(s),∫sti(τ)+1C(s,u,z(γi(τ)))du+∑k=i(τ)+1i(s+ω)−1∫tktk+1C(s,u,z(γk))du+∫ti(s+ω)s+ωC(s,u,z(γi(s+ω)))du)+g(s,z(s),z(γi(τ)))]ds +∑j=i(τ)+1i(t)−1∫tjtj+1Φ(t,s)×[f(s,z(s),∫stj+1C(s,u,z(γj))du+∑k=j+1i(s+ω)−1∫tktk+1C(s,u,z(γk))du+∫ti(s+ω)s+ωC(s,u,z(γi(s+ω)))du)+g(s,z(s),z(γj))]ds +∫ti(t)tΦ(t,s)[f(s,z(s),∫sti(t)+1C(s,u,z(γi(t)))du+∑k=i(t)+1i(s+ω)−1∫tktk+1C(s,u,z(γk))du+∫ti(s+ω)s+ωC(s,u,z(γi(s+ω)))du)+g(s,z(s),z(γi(t)))]ds.

On the other hand, one can easily see that

(12) ∫τtg(s,z(s),z(γ(s)))ds  =∫τti(τ)+1g(s,z(s),z(γi(τ)))ds   +∑j=i(τ)+1i(t)−1∫tjtj+1g(s,z(s),z(γj))ds   +∫ti(t)tg(s,z(s),z(γi(t)))ds.

Then, any solution of the DEPCAG system (6) with the initial condition *z*(*τ*) = *ξ* can be written as

(13) z(t)=Φ(t,τ)ξ +∫τtΦ(t,s)×[f(s,z(s),∫ss+ωC(s,u,z(γ(u)))du)+g(s,z(s),z(γ(s)))]ds, τ∈ℝ.

Amongst these solutions, that one will be *ω*-periodic, for which *z*(*τ*) = *ξ* = *z*(*τ* + *ω*); by the condition (*N*_*ω*_) and using (13) we get

(14) ξ=(I−Φ(τ+ω,τ))−1 ×∫ττ+ωΦ(τ+ω,s)×[f(s,z(s),∫ss+ωC(s,u,z(γ(u)))du)+g(s,z(s),z(γ(s)))]ds.

A substitution of (14) into (13) yields

(15) z(t)=Φ(t,τ)(I−Φ(τ+ω,τ))−1 ×{∫ττ+ωΦ(τ+ω,s)×[f(s,z(s),∫ss+ωC(s,u,z(γ(u)))du)+g(s,z(s),z(γ(s)))]ds} +∫τtΦ(t,s)[f(s,z(s),∫ss+ωC(s,u,z(γ(u)))du)+g(s,z(s),z(γ(s)))]ds.

It is easy to check the following identity using properties of the function Φ(*t*):

(16) Φ−1(τ)(I−Φ(τ+ω,τ))−1Φ(τ+ω)  =(Φ−1(τ+ω)Φ(τ)−I)−1.

It follows that

(17) z(t)=∫τtΦ(t)(I+(Φ−1(τ+ω)Φ(τ)−I)−1)Φ(s)−1×[f(s,z(s),∫ss+ωC(s,u,z(γ(u)))du)+g(s,z(s),z(γ(s)))]ds +∫tτ+ωΦ(t)(Φ−1(τ+ω)Φ(τ)−I)−1Φ−1(s)×[f(s,z(s),∫ss+ωC(s,u,z(γ(u)))du)+g(s,z(s),z(γ(s)))]ds.

In such a case the DEPCAG system (6) has *ω*-periodic solution *z*(*t*) given by the integral equation (17). Before studying the existence of solutions of integral equation (17) in the next section, firstly, we define Green's function for periodic solutions of the DEPCAG system (6).

**Definition 2**

Suppose that the condition (*N*_*ω*_) holds. For each *t*, *s* ∈ [*τ*, *τ* + *ω*], Green's function for (6) is given by

(18) $$\begin{array}{c} G\left(t,s\right)=\{\begin{array}{cc} \Phi \left(t\right)\left(I+D\right)\Phi^{-1}\left(s\right), & \tau \leq s\leq t\leq \tau +\omega ,\\ \Phi \left(t\right)D\Phi^{-1}\left(s\right), & \tau \leq t<s\leq \tau +\omega , \end{array} \end{array}$$

where Φ(*t*) is a fundamental solution of (7) and

(19) $$\begin{array}{c} D={\left({\left(\Phi^{-1}\left(\tau \right)\Phi \left(\tau +\omega \right)\right)}^{-1}-I\right)}^{-1}. \end{array}$$

We note that the condition (*N*_*ω*_) implies the existence of the matrix *D*.

To prove double *ω*-periodicity of Green's function *G*(*t*, *s*), we first give the following lemma.

**Lemma 3**

Suppose that the condition (*N*_*ω*_) holds. Let the matrix *D* be defined by (19), and then one has the following identities:

(20) (I+D)=(Φ−1(τ)Φ(τ+ω))−1D=(I−Φ−1(τ)Φ(τ+ω))−1,Φ(t)DΦ−1(t+ω)−Φ(t)DΦ−1(t)=I,(I+D)Φ−1(τ−ω)Φ(τ)=D.

For Lemma 3 we can prove an important property, double *ω*-periodicity, of Green's function *G*(*t*, *s*) to study after the existence of periodic solutions.

**Lemma 4**

Suppose that the condition (*N*_*ω*_) holds. Then Green's function *G*(*t*, *s*) is double *ω*-periodic; that is, *G*(*t* + *ω*, *s* + *ω*) = *G*(*t*, *s*).

## 3. Existence of Periodic Solutions

In this section, we prove the main theorems of this paper, so we recall the nonlinear integro-differential systems with DEPCAG (6):

(21) z′(t)=A(t)z(t)+f(t,z(t),∫tt+ωC(t,s,z(γ(s)))ds) +g(t,z(t),z(γ(t))), t∈ℝ.

For this, a natural Banach space is

(22) ℙω={ϕ:ℝ⟶ℝn ∣ ϕ  is  ω-periodiccontinuous  function}

with the supremum norm

(23) $$\begin{array}{c} ||z||=\underset{t\in \mathbb{R}}{\sup}\left|z\left(t\right)\right|=\underset{t\in \left[\tau ,\tau +\omega \right]}{\sup}\left|z\left(t\right)\right|. \end{array}$$

Consider *f* : ℝ × ℝ^*n*^ × ℝ^*n*^ → ℝ^*n*^ and *g* : ℝ × ℝ^*n*^ × ℝ^*n*^ → ℝ^*n*^ are continuous functions. Moreover, we will refer to the following specific conditions.

*Continuous Condition*

(*C*) Let *R* > 0, *t*, *s* ∈ ℝ and *y*_1_, *y*_2_ ∈ ℝ^*n*^, |*y*_*i*_| ≤ *R*, *i* = 1,2. For any *ϵ* > 0 there exist *δ* > 0 and *λ* : ℝ^2^ → [0, *∞*) a function such that |*y*_1_ − *y*_2_| ≤ *δ* implies
  (24) $$\begin{array}{c} \left|C\left(t,s,y_{1}\right)-C\left(t,s,y_{2}\right)\right|\leq \epsilon \lambda \left(t,s\right),\;t,s\in \mathbb{R}, \end{array}$$

where $\underset{}{\sup_{t\in \mathbb{R}}}\;\int_{t}^{t+\omega }\lambda (t,s)ds=\hat{\lambda }$.

*Lipschitz Conditions*

(*L*_*C*_) There exists a continuous function *λ* : ℝ^2^ → [0, *∞*) such that *t*, *s* ∈ ℝ and for any *y*_1_, *y*_2_ ∈ ℝ^*n*^ we have
  (25) $$\begin{array}{c} \left|C\left(t,s,y_{1}\right)-C\left(t,s,y_{2}\right)\right|\leq \lambda \left(t,s\right)\left|y_{1}-y_{2}\right|. \end{array}$$

Moreover, $\underset{}{\sup_{t\in \mathbb{R}}}\int_{t}^{t+\omega }\lambda (t,s)ds=\hat{\lambda }$ and $\underset{}{\sup_{t\in \mathbb{R}}}\int_{t}^{t+\omega }\left|C\left(t,s,0\right)\right|ds={\hat{\lambda }}_{0}$.

(*L*_*f*_) For *t* ∈ ℝ and *x*_1_, *y*_1_, *x*_2_, *y*_2_ ∈ ℝ^*n*^, there exist functions *p*_1_, *p*_2_ : ℝ → [0, *∞*) such that
  (26) |f(t,x1,y1)−f(t,x2,y2)|  ≤p1(t)|x1−x2|+p2(t)|y1−y2|.

Moreover, $\alpha ={\underset{}{\max}}_{t\in \mathbb{R}}\left|f\left(t,0,0\right)\right|$,

(27)  ∫ττ+ω[p1(s)+λ^p2(s)]ds≤L1, η1=λ^·maxt∈[τ,τ+ω]  |p2(t)|,  ω∈ℝ+,  τ∈ℝ.

(*L*_*g*_) For *t* ∈ ℝ and *x*_1_, *y*_1_, *x*_2_, *y*_2_ ∈ ℝ^*n*^, there exist functions *υ*_1_, *υ*_2_ : ℝ → [0, *∞*) such that
  (28) |g(t,x1,y1)−g(t,x2,y2)|  ≤υ1(t)|x1−x2|+υ2(t)|y1−y2|.
 Moreover, $\beta ={\underset{}{\max}}_{t\in \mathbb{R}}\left|g\left(t,0,0\right)\right|$ and
  (29) $$\begin{array}{c} \overset{\tau +\omega }{\underset{\tau }{\int }}\left[\upsilon_{1}\left(s\right)+\upsilon_{2}\left(s\right)\right]ds\leq L_{2},\;\omega \in {\mathbb{R}}_{+},\;\;\tau \in \mathbb{R}. \end{array}$$

*Invariance Conditions*

(*M*_*C*_) For every *R* > 0, *t*, *s* ∈ ℝ, |*y*| ≤ *R*, there exist *λ*,  *h* : ℝ^2^ → [0, *∞*) functions and positive constants $\hat{\lambda }$, $\hat{h}$ for which
  (30) $$\begin{array}{c} \left|C\left(t,s,y\right)\right|\leq \lambda \left(t,s\right)\left|y\right|+h\left(t,s\right), \end{array}$$

where $\underset{}{\sup_{t\in \mathbb{R}}}\int_{t}^{t+\omega }\lambda (t,s)ds=\hat{\lambda }$ and $\underset{}{\sup_{t\in \mathbb{R}}}\int_{t}^{t+\omega }h(t,s)ds=\hat{h}$.

(*M*_*f*_) For every *R* > 0, *t* ∈ ℝ, |*x*|, |*y*| ≤ *R*, there exist functions *m*_1_, *m*_2_ : ℝ → [0, *∞*) and positive constants *ρ*_1_,  *C*_1_,  *η*_2_ for which
  (31) $$\begin{array}{c} \left|f\left(t,x,y\right)\right|\leq m_{1}\left(t\right)\left|x\right|+m_{2}\left(t\right)\left|y\right|+\rho_{1}, \end{array}$$

where $\int_{\tau }^{\tau +\omega }\left[m_{1}(s)+\hat{\lambda }m_{2}(s)\right]ds\leq C_{1}$ and $\eta_{2}=\hat{h}\cdot \underset{}{\max_{t\in [\tau ,\tau +\omega ]}}\;\left|m_{2}(t)\right|,\omega \in {\mathbb{R}}_{+},\tau \in \mathbb{R}$.

(*M*_*g*_) For every *R* > 0, *t* ∈ ℝ, |*x*|, |*y*| ≤ *R*, there exist functions *κ*_1_,  *κ*_2_ : ℝ → [0, *∞*) and positive constants *ρ*_2_,  *C*_2_ for which
  (32) $$\begin{array}{c} \left|g\left(t,x,y\right)\right|\leq \kappa_{1}\left(t\right)\left|x\right|+\kappa_{2}\left(t\right)\left|y\right|+\rho_{2}, \end{array}$$

where ∫_*τ*_^*τ*+*ω*^[*κ*_1_(*s*) + *κ*_2_(*s*)]*ds* ≤ *C*_2_, *ω* ∈ ℝ_+_, *τ* ∈ ℝ.

*Periodic Conditions*

(*P*) There exists *ω* > 0 such that
  (1) *A*(*t*), *f*(*t*, *x*_1_, *y*_1_), and *g*(*t*, *x*_2_, *y*_2_) are periodic functions in *t* with a period *ω* for all *t* ≥ *τ*;
  (2) *C*(*t* + *ω*, *s* + *ω*, *x*_1_) = *C*(*t*, *s*, *x*_1_) for all *t* ≥ *τ*;
  (3) there exists *p* ∈ *ℤ* ^+^, for which the sequences {*t*_*i*_}_*i*∈*ℤ*_, {*γ*_*i*_}_*i*∈*ℤ*_ satisfy the (*ω*, *p*) condition; that is,
    (33) $$\begin{array}{c} t_{i+p}=t_{i}+\omega ,\;\;\gamma_{i+p}=\gamma_{i}+\omega ,\;\text{for}\;\;i\in \mathbb{Z}. \end{array}$$

**Remark 5**

Note that (*ω*, *p*) condition is a discrete relation, which moves the interval *I*_*i*_ into *I*_*i*+*p*_. Then we have the following consequences.

(i) For any *τ* ∈ ℝ, the interval [*τ*, *τ* + *ω*] can be decomposed as follows:
  (34) $$\begin{array}{c} \left[\tau ,t_{i(\tau )+1}\right]\cup \overset{i(\tau )+p-1}{\underset{j=i\left(\tau \right)+1}{⋃}}I_{j}\cup \left[t_{i(\tau )+p},\tau +\omega \right]. \end{array}$$
(ii) For *t* ∈ [*t*_*i*_, *t*_*i*+1_), we have
  (35) $$\begin{array}{c} \left(\text{a}\right)\;\;t+\omega \in \left[t_{i+p},t_{i+p+1}\right),\;\;\left(\text{b}\right)\;\;\gamma \left(t\right)+\omega \in \left[t_{i+p},t_{i+p+1}\right). \end{array}$$

Then,

(36) $$\begin{array}{c} \gamma \left(t+\omega \right)=\gamma_{i(t+\omega )}=\gamma_{i(t)+p}=\gamma_{i(t)}+\omega =\gamma \left(t\right)+\omega . \end{array}$$

Using Definition 2, Remark 5, and double *ω*-periodicity of Green's function, similar formula is given by (17). So, we have obtained the following result.

**Proposition 6**

Suppose that the conditions (*N*_*ω*_) and (P) hold. Let (*τ*, *z*(*τ*)) ∈ ℝ × ℝ^*n*^. Then, *z*(*t*) = *z*(*t*, *τ*, *z*(*τ*)) is *ω*-periodic solution on ℝ of the DEPCAG system (6) if and only if *z*(*t*) is *ω*-periodic solution of the integral equation

(37) z(t)=∫ττ+ωG(t,s)[f(s,z(s),∫ss+ωC(s,u,z(γ(u)))du)+g(s,z(s),z(γ(s)))]ds,

where Green's function *G*(*t*, *s*) is defined by (18).

Consider the operator *ℑ* : *ℙ*_*ω*_ → *ℙ*_*ω*_ by

(38) (ℑz)(t)=∫ττ+ωG(t,s)×[f(s,z(s),∫ss+ωC(s,u,z(γ(u)))du)+g(s,z(s),z(γ(s)))]ds.

It is easy to see that the DEPCAG system (6) has *ω*-periodic solution if and only if the operator *ℑ* has one fixed point in *ℙ*_*ω*_.

To prove some existence criteria for *ω*-periodic solutions of the DEPCAG system (6) we use the Banach, Schauder, and Krasnoselskii's fixed point theorems.

Next we state first Krasnoselskii's fixed point theorem which enables us to prove the existence of a periodic solution. For the proof of Krasnoselskii's fixed point theorem we refer the reader to [43].

**Theorem A (Krasnoselskii's fixed point theorem)**

Let *S* be a closed convex nonempty subset of a Banach space (*E*, ||·||). Suppose that *𝒜* and *ℬ* map *S* into *E* such that

- *x*,  *y* ∈ *S*, implies *𝒜x* + *ℬy* ∈ *S*,
- *𝒜* is a contraction mapping,
- *ℬ* is completely continuous.

Then there exists *z* ∈ *S* with *z* = *𝒜z* + *ℬz*.

**Remark 7**

Krasnoselskii's theorem may be combined with Banach and Schauder's fixed point theorems. In a certain sense, we can interpret this as follows: if a compact operator has the fixed point property, under a small perturbation, then this property can be inherited. The theorem is useful in establishing the existence results for perturbed operator equations. It also has a wide range of applications to nonlinear integral equations of mixed type for proving the existence of solutions. Thus the existence of fixed points for the sum of two operators has attracted tremendous interest, and their applications are frequent in nonlinear analysis. See [32, 33, 43–46].

We note that to apply Krasnoselskii's fixed point theorem we need to construct two mappings; one is contraction and the other is compact. Therefore, we express (38) as

(39) $$\begin{array}{c} \left(ℑz\right)\left(t\right)=\left(𝒜z\right)\left(t\right)+\left(ℬz\right)\left(t\right), \end{array}$$

where *𝒜*, *ℬ* : *ℙ*_*ω*_ → *ℙ*_*ω*_ are given by

(40) (𝒜z)(t)  =∫ττ+ωG(t,s)f(s,z(s),∫ss+ωC(s,u,z(γ(u)))du)ds,

(41) $$\begin{array}{c} \left(ℬz\right)\left(t\right)=\overset{\tau +\omega }{\underset{\tau }{\int }}G\left(t,s\right)g\left(s,z\left(s\right),z\left(\gamma \left(s\right)\right)\right)ds. \end{array}$$

To simplify notations, we introduce the following constant and sets:

(42) $$\begin{array}{c} c_{G}=\underset{t,s\in \left[\tau ,\tau +\omega \right]}{\max}\left|G\left(t,s\right)\right|,\;\;𝕊=\left\{z\in {\mathbb{P}}_{\omega }:||z||\leq R\right\},\\ {𝒞}_{ℛ}=\left\{z\in {\mathbb{R}}^{n}:||z||\leq R\right\}. \end{array}$$

**Lemma 8**

If (*N*_*ω*_), (*P*), (*L*_*f*_), and (*L*_*C*_) hold, *𝒜* is given by (40) with *c*_*G*_ *L*_1_ < 1, and then *𝒜* is a contraction mapping.

**Proof**

Let *𝒜* be defined by (40). First we want to show that (*𝒜φ*)(*t* + *ω*) = (*𝒜φ*)(*t*).

Let *φ* ∈ *ℙ*_*ω*_. Then using (40), the periodicity of Green's function, and (*ω*, *p*) condition, we arrive at

(43) (𝒜φ)(t+ω) =∫τ+ωτ+2ωG(t+ω,s)×f(s,φ(s),∫ss+ωC(s,u,φ(γ(u)))du)ds =∫ττ+ωG(t+ω,s+ω)×f(s+ω,φ(s+ω),∫s+ωs+2ωC(s+ω,u,φ(γ(u)))du)ds =∫ττ+ωG(t+ω,s+ω)×f(s+ω,φ(s+ω),∫ss+ωC(s+ω,u+ω,φ(γ(u+ω)))du)ds =∫ττ+ωG(t+ω,s+ω)×f(s+ω,φ(s+ω),∫ss+ωC(s+ω,u+ω,φ(γ(u)+ω))du)ds =∫ττ+ωG(t,s)×f(s,φ(s),∫ss+ωC(s,u,φ(γ(u)))du)ds =(𝒜φ)(t).

Secondly, we show that *𝒜* is a contraction mapping. Let *φ*,  *ζ* ∈ *ℙ*_*ω*_; then we have

(44) ||𝒜φ−𝒜ζ|| =supt∈[τ,τ+ω]|𝒜φ(t)−𝒜ζ(t)| ≤supt∈[τ,τ+ω]∫ττ+ω|G(t,s)|×|f(s,φ(s),∫ss+ωC(s,u,φ(γ(u)))du)−f(s,ζ(s),∫ss+ωC(s,u,ζ(γ(u)))du)|ds ≤cG∫ττ+ω[p1(s)|φ(s)−ζ(s)|+p2(s)×|∫ss+ωC(s,u,φ(γ(u)))du−∫ss+ωC(s,u,ζ(γ(u)))du|]ds ≤cG∫ττ+ω[p1(s)|φ(s)−ζ(s)|+p2(s)∫ss+ωλ(s,u)×|φ(γ(u))−ζ(γ(u))|du]ds ≤(cG∫ττ+ω[p1(s)+p2(s)∫ss+ωλ(s,u)du]ds)  ×||φ−ζ|| ≤(cG∫ττ+ω[p1(s)+λ^p2(s)]ds)||φ−ζ|| ≤cGL1||φ−ζ||.

Hence *𝒜* defines a contraction mapping.

Similarly, *ℬ* is given by (41), which may be also a contraction operator.

**Lemma 9**

If (*N*_*ω*_), (*P*), and (*L*_*g*_) hold, *ℬ* is given by (41) with *c*_*G*_ *L*_2_ < 1, and then *ℬ* is a contraction mapping.

**Lemma 10**

If (*N*_*ω*_) holds, *ℬ* is defined by (41), and then *ℬ* is completely continuous; that is, *ℬ* is continuous and the image of *ℬ* is contained in a compact set.

**Proof**

*Step  1*. First we prove that *ℬ* : *ℙ*_*ω*_ → *ℙ*_*ω*_ is continuous.

As the operator *𝒜*, a change of variable in (41), we have (*ℬφ*)(*t* + *ω*) = (*ℬφ*)(*t*). Now, we want to show *ℬ* is continuous.

The function *g*(*t*, *x*, *y*) is uniformly continuous on [*τ*, *τ* + *ω*] × *𝒞*_*ℛ*_ × *𝒞*_*ℛ*_ and by the periodicity in *t*, the function *g*(*t*, *x*, *y*) is uniformly continuous on ℝ × *𝒞*_*ℛ*_ × *𝒞*_*ℛ*_. Thus, for any *ϵ*′ = (*ϵ*/*c*_*G*_ *ω*) > 0, there exists *δ* = *δ*(*ϵ*) > 0 such that *z*_1_, *z*_2_ ∈ *𝕊*, ||*z*_1_ − *z*_2_|| ≤ *δ* implies |*g*(*t*, *z*_1_(*t*), *z*_1_(*γ*(*t*))) − *g*(*t*, *z*_2_(*t*), *z*_2_(*γ*(*t*)))| ≤ *ϵ*′ for *t* ∈ [*τ*, *τ* + *ω*]. Then ||*ℬz*_1_ − *ℬz*_2_|| ≤ *ϵ*. In fact, by the continuity of *g*,

(45) |g(t,z1(t),z1(γ(t)))−g(t,z2(t),z2(γ(t)))|≤ϵ′for  t∈[τ,τ+ω]

and then

(46) ||ℬz1−ℬz2|| =supt∈[τ,τ+ω]|ℬz1(t)−ℬz2(t)| ≤supt∈[τ,τ+ω]∫ττ+ω|G(t,s)||g(s,z1(s),z1(γ(s)))−g(s,z2(s),z2(γ(s)))|ds ≤∫ττ+ωcGϵ′ds≤cGϵ′ω=ϵ,

and the continuity of *ℬ* is proved.

*Step  2*. We show that the image of *ℬ* is contained in a compact set.

Let *x*, *y* ∈ *𝒞*_*ℛ*_ and *s* ∈ [*τ*, *τ* + *ω*]; for the continuity of the function *g*(*s*, *x*, *y*), there exists *M* > 0 such that |*g*(*s*, *x*, *y*)| ≤ *M*. Let *φ*_*n*_ ∈ *𝕊* where *n* is a positive integer; then we have

(47) ||ℬφn||=supt∈[τ,τ+ω]|ℬφn(t)|≤supt∈[τ,τ+ω]∫ττ+ω|G(t,s)||g(s,φn(s),φn(γ(s)))|ds≤cG∫ττ+ω|g(s,φn(s),φn(γ(s)))|ds≤cGMω.

Moreover, a direct calculation (*ℬφ*_*n*_(*t*))′ shows that

(48) (ℬφn(t))′ =(∫ττ+ωG(t,s)g(s,φn(s),φn(γ(s)))ds)′ =Φ′(t)∫τt(I+D)Φ−1(s)g(s,φn(s),φn(γ(s)))ds  +Φ(t)(I+D)Φ−1(t)g(t,φn(t),φn(γ(t)))  +Φ′(t)∫tτ+ω[DΦ−1(s)g(s,φn(s),φn(γ(s)))]ds  −Φ(t)DΦ−1(t)g(t,φn(t),φn(γ(t))) =A(t)(∫ττ+ωG(t,s)g(s,φn(s),φn(γ(s)))ds)  +g(t,φn(t),φn(γ(t))) =A(t)ℬφn(t)+g(t,φn(t),φn(γ(t))).

As *A*(*t*) is bounded on [*τ*, *τ* + *ω*] and *ℬφ*_*n*_(*t*), *g*(*t*, *φ*_*n*_(*t*), *φ*_*n*_(*γ*(*t*)) are bounded on [*τ*, *τ* + *ω*] × *𝕊* × *𝕊*. Thus, the above expression yields ||(*ℬφ*_*n*_)′|| ≤ *L*, for some positive constant *L*. Hence the sequence (*ℬφ*_*n*_) is uniformly bounded and equicontinuous. Ascoli-Arzela's theorem implies that a subsequence (*ℬφ*_*n*_*k*__) of (*ℬφ*_*n*_) converges uniformly to a continuous *ω*-periodic function. Thus *ℬ* is continuous and *ℬ*(*𝕊*) is a compact set.

In a similar way, for *𝒜* we obtain the following.

**Lemma 11**

If (*N*_*ω*_) and (*C*) hold, *𝒜* is defined by (40), and then *𝒜* is completely continuous.

**Theorem 12**

Suppose the hypotheses (*N*_*ω*_), (*P*), (*L*_*f*_), (*L*_*C*_), (*M*_*g*_) hold. Let *R* be a positive constant satisfying the inequality

(49) $$\begin{array}{c} c_{G}\left(L_{1}+C_{2}\right)R+c_{G}\left(\alpha +\eta_{1}+\rho_{2}\right)\omega \leq R. \end{array}$$

Then the DEPCAG system (6) has at least one *ω*-periodic solution in *𝕊*.

**Proof**

By Lemma 8, the mapping *𝒜* is a contraction and it is clear that *𝒜* : *ℙ*_*ω*_ → *ℙ*_*ω*_. Also, from Lemma 10, *ℬ* is completely continuous.

Next, we prove that if *φ*,  *ζ* ∈ *𝕊* with ||*φ*|| ≤ *R* and ||*ζ*|| ≤ *R*, then ||*𝒜ϕ* + *ℬζ*|| ≤ *R*.

Let *φ*,  *ζ* ∈ *𝕊* with ||*φ*|| ≤ *R* and ||*ζ*|| ≤ *R*. Then

(50) ||𝒜φ+ℬζ|| =supt∈[τ,τ+ω]|∫ττ+ωG(t,s)×[f(s,φ(s),∫ss+ωC(s,u,φ(γ(u)))du)−f(s,0,0)+f(s,0,0)]ds+∫ττ+ωG(t,s)g(s,ζ(s),ζ(γ(s)))ds| ≤supt∈[τ,τ+ω]  ∫ττ+ω|G(t,s)|×|f(s,φ(s),∫ss+ωC(s,u,φ(γ(u)))du)−f(s,0,0)|ds  +supt∈[τ,τ+ω]∫ττ+ω|G(t,s)||f(s,0,0)|ds  +supt∈[τ,τ+ω]∫ττ+ω|G(t,s)||g(s,ζ(s),ζ(γ(s)))|ds ≤cG∫ττ+ωp1(s)|φ(s)|+p2(s)|∫ss+ωC(s,u,φ(γ(u)))du|ds  +αcGω+cG(∫ττ+ω[κ1(s)+κ1(s)]ds)||ζ||+ρ2cGω ≤cG∫ττ+ωp1(s)|φ(s)|+p2(s)×|∫ss+ω[C(s,u,φ(γ(u)))−C(s,u,0)+C(s,u,0)]du|ds  +αcGω+cG(∫ττ+ω[κ1(s)+κ1(s)]ds)||ζ||+ρ2cGω ≤cG∫ττ+ωp1(s)|φ(s)|+p2(s)×|∫ss+ω[λ(s,u)|φ(γ(u))|+|C(s,u,0)|]du|ds  +αcGω+cG(∫ττ+ω[κ1(s)+κ1(s)]ds)||ζ||+ρ2cGω ≤cG(∫ττ+ωp1(s)+λ^p2(s)ds)||φ||  +cG(∫ττ+ω[κ1(s)+κ1(s)]ds)||ζ||  +cGλ^0∫ττ+ωp2(s)ds+αcGω+ρ2cGω ≤cG(L1+C2)R+cG(α+η1+ρ2)ω.

We now see that all the conditions of Krasnoselskii's theorem are satisfied. Thus there exists a fixed point *z* in *𝕊* such that *z* = *𝒜z* + *ℬz*. By Proposition 6, this fixed point is a solution of the DEPCAG system (6). Hence the DEPCAG system (6) has *ω*-periodic solution.

By the symmetry of the conditions, we will obtain as Theorem 12.

**Theorem 13**

Suppose the hypothesis (*N*_*ω*_), (*P*), (*L*_*g*_), (*M*_*f*_), (*M*_*C*_), (*C*) hold. Let *R* be a positive constant satisfying the inequality

(51) $$\begin{array}{c} c_{G}\left(C_{1}+L_{2}\right)R+c_{G}\left(\beta +\eta_{2}+\rho_{1}\right)\omega \leq R. \end{array}$$

Then the DEPCAG system (6) has at least one *ω*-periodic solution in *𝕊*.

By Lemma 9, the mapping *ℬ* is a contraction and it is clear that *ℬ* : *ℙ*_*ω*_ → *ℙ*_*ω*_. Also, from Lemma 11, *𝒜* is completely continuous.

Next, we prove that if *φ*,  *ζ* ∈ *𝕊* with ||*φ*|| ≤ *R* and ||*ζ*|| ≤ *R*, then ||*𝒜ϕ* + *ℬζ*|| ≤ *R*.

Let *φ*,  *ζ* ∈ *𝕊* with ||*φ*|| ≤ *R* and ||*ζ*|| ≤ *R*. Then

(52) ||𝒜φ+ℬζ||=supt∈[τ,τ+ω]|∫ττ+ωG(t,s)×f(s,φ(s),∫ss+ωC(s,u,φ(γ(u)))du)ds+∫ττ+ωG(t,s)[(g(s,ζ(s),ζ(γ(s)))−g(s,0,0)+g(s,0,0))]ds|≤supt∈[τ,τ+ω]∫ττ+ω|G(t,s)|×(m1(t)|φ(s)|+m2(t)×|∫ss+ωC(s,u,φ(γ(u)))du|+ρ1)ds +supt∈[τ,τ+ω]∫ττ+ω|G(t,s)|×(υ1(s)ζ(s)+υ2(s)ζ(γ(s)))ds +supt∈[τ,τ+ω]∫ττ+ω|G(t,s)||g(s,0,0)|ds≤cG∫ττ+ω(m1(t)|φ(s)|+m2(t)×|∫ss+ω[λ(s,u)|φ(γ(u))|+|h(s,u)|]du|+ρ1)ds +cG(∫ττ+ω(υ1(s)+υ2(s))ds)||ζ||+βcGω≤cG(∫ττ+ω[m1(s)+λ^m2(s)]ds)||φ|| +cGh^∫ττ+ωm2(s)ds+ρ1cGω +cG(∫ττ+ω(υ1(s)+υ2(s))ds)||ζ||+βcGω≤cG(C1+L2)R+cG(β+η2+ρ1)ω.

Applying Banach's fixed point theorem we have the following.

**Theorem 14**

Suppose the hypotheses (*N*_*ω*_), (*P*), (*L*_*f*_), (*L*_*C*_), (*L*_*g*_) hold. If

(53) $$\begin{array}{c} c_{G}\left(L_{1}+L_{2}\right)<1, \end{array}$$

then the DEPCAG system (6) has a unique *ω*-periodic solution.

**Proof**

Let the mapping *ℑ* be given by (38). For *φ*,  *ζ* ∈ *ℙ*_*ω*_, in view of (38), we have

(54) ||ℑφ−ℑζ||≤supt∈[τ,τ+ω]∫ττ+ω|G(t,s)|×|f(s,φ(s),∫ss+ωC(s,u,φ(γ(u)))du)−f(s,ζ(s),∫ss+ωC(s,u,ζ(γ(u)))du)|ds +supt∈[τ,τ+ω]∫ττ+ω|G(t,s)||g(s,φ(s),φ(γ(s)))−g(s,ζ(s),ζ(γ(s)))|ds≤cG∫ττ+ω[p1(s)|φ(s)−ζ(s)|+p2(s)×|∫ss+ωC(s,u,φ(γ(u)))du−∫ss+ωC(s,u,ζ(γ(u)))du|]ds +cG∫ττ+ω|g(s,φ(s),φ(γ(s)))−g(s,ζ(s),ζ(γ(s)))|ds≤cG(∫ττ+ω[p1(s)+λ^p2(s)]ds+∫ττ+ω[υ1(s)+υ2(s)]ds)||φ−ζ||≤cG(L1+L2)||φ−ζ||.

This completes the proof by invoking the contraction mapping principle.

As a direct consequence of the method, Schauder's theorem implies the following.

**Theorem 15**

Suppose the hypotheses (*N*_*ω*_), (*P*), (*M*_*f*_), (*M*_*C*_), (*M*_*g*_), (*C*) hold. Let *R* be a positive constant satisfying the inequality

(55) $$\begin{array}{c} c_{G}\left(C_{1}+C_{2}\right)R+c_{G}\left(\rho_{1}+\rho_{2}+\eta_{2}\right)\omega \leq R. \end{array}$$

Then the DEPCAG system (6) has at least one *ω*-periodic solution in *𝕊*.

**Remark 16**

Considering the DEPCAG system (6)

(56) $$\begin{array}{c} x^{\prime }\left(t\right)=A\left(t\right)x\left(t\right)+g\left(t,x\left(t\right),x\left(\gamma \left(t\right)\right)\right). \end{array}$$

Krasnoselskii (see [47]) proved that if *A* is a stable constant matrix, without piecewise alternately advanced and retarded argument and lim_|*x*|+|*y*|→+*∞*_  (|*g*(*t*, *x*, *y*)|/(|*x*| + |*y*|)) = 0, then the system (56) has at least one periodic solution. In our case, applying Theorem 12, this result is also valid for the DEPCAG system (56), requiring the hypothesis: (*N*_*ω*_), (*P*_3_), (*M*_*g*_), *A*(*t*) and *g*(*t*, *x*, *y*) are periodic functions in *t* with a period *ω* for all *t* ≥ *τ* and |*g*(*t*, *x*, *y*)| ≤ *c*_1_(|*x*| + |*y*|) + *ρ*_2_, with 2*c*_1_ *ω* < *C*_2_ and *ρ*_2_ constants for |*x*| + |*y*| ≤ *R*, *R* > 0.

**Remark 17**

Considering the nonlinear system of differential equations with a general piecewise alternately advanced and retarded argument,

(57) x′(t)=A(t)x(t)+f(t,x(t),x(γ(t))) +g(t,x(t),x(γ(t))).

In this particular case, applying Theorem 13, this result is also valid for the DEPCAG system (57), requiring the hypothesis: (*N*_*ω*_), (*P*_1_), (*P*_3_), (*L*_*g*_), (*M*_*f*_), and *c*_*G*_(*C*_1_ + *L*_2_)*R* + *c*_*G*_(*β* + *ρ*_1_)*ω* ≤ *R* with $\hat{\lambda }=1$.

**Remark 18**

Suppose that (*P*_1_) is satisfied by *ω* = *ω*_1_, (*P*_2_) by *ω* = *ω*_2_, and (*P*_3_) by *ω* = *ω*_3_, if *ω*_*i*_/*ω*_*j*_ is a rational number for all *i*, *j* = 1,2, 3; then (*P*_1_), (*P*_2_), and (*P*_3_) are simultaneously satisfied by *ω* = l.c.m.{*ω*_1_, *ω*_2_, *ω*_3_}, where l.c.m.{*ω*_1_, *ω*_2_, *ω*_3_} denotes the least common multiple between *ω*_1_, *ω*_2_ and *ω*_3_. In the general case it is possible that there exist five possible periods: *ω*_1_ for *A*, *ω*_2_ for *f*, *ω*_3_ for *g*, *ω*_4_ for *C*, and the sequences {*t*_*i*_}_*i*∈*ℤ*_, {*γ*_*i*_}_*i*∈*ℤ*_ satisfy the (*ω*_5_, *p*) condition. If *ω*_*i*_/*ω*_*j*_ is a rational number for all *i*, *j* = 1,2, 3,4, 5, so, in this situation our results insure the existence of *ω*-periodic solution with *ω* = l.c.m.{*ω*_1_, *ω*_2_, *ω*_3_, *ω*_4_, *ω*_5_}. Therefore the above results insure the existence of *ω*-periodic solutions of the DEPCAG system (6). These solutions are called subharmonic solutions. See Corollaries 19–22.

To determine criteria for the existence and uniqueness of subharmonic solutions of the DEPCAG system (6), from now on we make the following assumption.

(*P*_*ω*_) There exists *ω* = l.c.m.{*ω*_1_, *ω*_2_, *ω*_3_, *ω*_4_, *ω*_5_} > 0, *ω*_*i*_/*ω*_*j*_ which is a rational number for all *i*, *j* = 1,2, 3,4, 5 such that
  1. *A*(*t*), *f*(*t*, *x*_1_, *y*_1_), and *g*(*t*, *x*_2_, *y*_2_) are periodic functions in *t* with a period *ω*_1_, *ω*_2_, and *ω*_3_, respectively, for all *t* ≥ *τ*;
  2. *C*(*t* + *ω*_4_, *s* + *ω*_4_, *x*_3_) = *C*(*t*, *s*, *x*_3_), for all *t* ≥ *τ*;
  3. There exists *p* ∈ *ℤ* ^+^, for which the sequences {*t*_*i*_}_*i*∈*ℤ*_, {*γ*_*i*_}_*i*∈*ℤ*_ satisfy the (*ω*_5_, *p*) condition.

As immediate corollaries of Theorems 12–15 and Remark 18, the following results are true.

**Corollary 19**

Suppose the hypotheses (*N*_*ω*_), (*P*_*ω*_), (*L*_*f*_), (*L*_*C*_), (*M*_*g*_) and (49) hold. Then the DEPCAG system (6) has at least one subharmonic solution in *𝕊*.

**Corollary 20**

Suppose the hypotheses (*N*_*ω*_), (*P*_*ω*_), (*L*_*g*_), (*M*_*f*_), (*M*_*C*_), (*C*) and (51) hold. Then the DEPCAG system (6) has at least one subharmonic solution in *𝕊*.

**Corollary 21**

Suppose the hypotheses (*N*_*ω*_), (*P*_*ω*_), (*L*_*f*_), (*L*_*C*_), (*L*_*g*_) and (53) hold. Then, the DEPCAG system (6) has a unique subharmonic solution.

**Corollary 22**

Suppose the hypotheses (*N*_*ω*_), (*P*_*ω*_), (*M*_*f*_), (*M*_*C*_), (*M*_*g*_), (*C*) and (55) hold. Then the DEPCAG system (6) has at least one subharmonic solution in *𝕊*.

## 4. Applications and Illustrative Examples

We will introduce appropriate examples in this section. These examples will show the feasibility of our theory.

Mathematical modelling of real-life problems usually results in functional equations, like ordinary or partial differential equations, integral and integro-differential equations, and stochastic equations. Many mathematical formulations of physical phenomena contain integro-differential equations; these equations arise in many fields like fluid dynamics, biological models, and chemical kinetics. So, we first consider nonlinear integro-differential equations with a general piecewise constant argument mentioned in the introduction and obtain some new sufficient conditions for the existence of the periodic solutions of these systems.

**Example 1**

Let *λ* : ℝ^2^ → [0, *∞*) and *h* : ℝ^2^ → [0, *∞*) be two functions satisfying

(58) $$\begin{array}{c} \underset{t\in \mathbb{R}}{\sup}\overset{t+2\pi }{\underset{t}{\int }}\lambda \left(t,s\right)ds\leq \hat{\lambda },\;\;\underset{t\in \mathbb{R}}{\sup}\overset{t+2\pi }{\underset{t}{\int }}h\left(t,s\right)ds\leq \hat{h}. \end{array}$$

Consider the following nonlinear integro-differential equations with piecewise alternately advanced and retarded argument of generalized type:

(59) z′(t)=a(t)z(t) +∫tt+2πln[1+|z(γ(s))|3λ(t,s)+h(t,s)]ds +(sint)z4(t)+(1+cos2t)z9(γ(t)), t∈ℝ,

where the sequences {*t*_*i*_}_*i*∈*ℤ*_ and {*γ*_*i*_}_*i*∈*ℤ*_ satisfy the (2*π*, *p*) condition, *λ* and *h* are double 2*π*-periodic continuous functions, and *a* is a 2*π*-periodic continuous function satisfying (*N*_*ω*_).

The conditions of Theorem 15 are fulfilled. Indeed,

- *f*(*t*, *x*, *y*) = *y* satisfies (*M*_*f*_): |*f*(*t*, *x*, *y*)|≤|*y*|;
- *g*(*t*, *x*, *y*) = (sin*t*)*x* ^4^ + (1 + cos^2^ *t*)*y* ^9^ satisfies (*M*_*g*_): |*g*(*t*, *x*, *y*)| ≤ *c*_1_(*R*)(|*x*| + |*y*|) + *ρ*_2_ for every *x*, *y* such that |*x*|, |*y*| ≤ *R*, uniformly in *t* ∈ ℝ where 4*πc*_1_(*R*) ≤ *C*_2_;
- *C*(*t*, *s*, *y*) = ln[1 + |*y* ^3^|*λ*(*t*, *s*) + *h*(*t*, *s*)] satisfies (*M*_*C*_): |*C*(*t*, *s*, *y*)| ≤ *c*_2_(*R*)*λ*(*t*, *s*)|*y*| + *h*(*t*, *s*) for |*y*| ≤ *R*, where $2\pi \hat{\lambda }\leq C_{1}$;
- *g* : ℝ × ℝ^*n*^ × ℝ^*n*^ → ℝ^*n*^ is continuous and *C*(*t*, *s*, *y*) satisfies (*C*).

Indeed, for any *ɛ* > 0, there exists *δ* > 0 such that |*y*_1_ − *y*_2_| ≤ *δ* implies

(60) $$\begin{array}{c} \left|C\left(t,s,y_{1}\right)-C\left(t,s,y_{2}\right)\right|\leq ɛc_{2}\left(R\right)\lambda \left(t,s\right)\;\text{for}\;\;t,s\in \mathbb{R}. \end{array}$$

Furthermore, there exists *R* such that

(61) $$\begin{array}{c} \frac{\exp\left(2a^{*}\pi \right)}{\exp\left(2a_{*}\pi \right)-1}\left[\left(C_{1}+C_{2}\right)R+2\pi \left(\rho_{2}+\hat{h}\right)\right]\leq R, \end{array}$$

where a* = sup_*t*∈ℝ_  |*a*(*t*)| and *a*_∗_ = inf_*t*∈ℝ_|*a*(*t*)|.

Then, by Theorem 15, the DEPCAG system (59) has at least one 2*π*-periodic solution.

**Example 2**

Thus many examples can be constructed where our results can be applied.

Let Λ : ℝ → ℝ^*n*×*n*^ and *μ* : ℝ → ℝ^*n*^ be two functions satisfying

(62) $$\begin{array}{c} \underset{t\in \mathbb{R}}{\sup}\overset{t+\omega }{\underset{t}{\int }}\left|\Lambda \left(s\right)\right|ds=\hat{\Lambda }<\infty ,\\ \underset{t\in \mathbb{R}}{\sup}\overset{t+\omega }{\underset{t}{\int }}\left|\mu \left(t-s\right)\right|ds=\hat{\mu }<\infty . \end{array}$$

Now, consider the integro-differential system with piecewise alternately advanced and retarded argument

(63) z′(t)=A(t)z(t)+B(t)g(z(t),z(γ(t))) +∫tt+ω[Λ(s)κ(z(γ(s)))+μ(t−s)]ds,

where the sequences {*t*_*i*_}_*i*∈*ℤ*_ and {*γ*_*i*_}_*i*∈*ℤ*_ satisfy the (*ω*, *p*) condition, *A*, *B*, Λ, *κ*, and *μ* are *ω*-periodic continuous functions, and

- *z*′(*t*) = *A*(*t*)*z*(*t*) satisfies (*N*_*ω*_);
- *B* is *ω*-periodic matrix function: |*B*(*t*)| ≤ *b*, *g* : ℝ^*n*^ × ℝ^*n*^ → ℝ^*n*^ is a continuous function, and |*g*(*x*, *y*)| ≤ *c*_1_(*R*)|*x*| + *c*_2_(*R*)|*y*| + *ϑ*_1_ for |*x*|, |*y*| ≤ *R*, where (*c*_1_(*R*) + *c*_1_(*R*))*ω* ≤ *C*_2_;
- |*κ*(*x*) − *κ*(*y*)| ≤ *c*_3_(*R*)|*x* − *y*|, where $c_{3}(R)\hat{\Lambda }\omega \leq L_{1}$.

The hypotheses of Theorem 12 are fulfilled. Then if there exists *R* such that

(64) $$\begin{array}{c} C_{G}\left(L_{1}+bC_{2}\right)R+C_{G}\left(b\vartheta_{1}+\kappa \left(0\right)\hat{\Lambda }+\hat{\mu }\right)\omega <R, \end{array}$$

Theorem 12 implies that there exists at least a *ω*-periodic solution of the DEPCAG system (63).

Note that similar results can be obtained under (*L*_*g*_) and (*M*_*C*_). On the other hand, the periodic situation of the DEPCAG system (56) and (57) can be treated in the same way.

Let us consider another example for second-order differential equations with a general piecewise constant argument. In this case, we can show the existence and uniqueness of periodic solutions of the following nonlinear DEPCAG system.

**Example 3**

Consider the following nonlinear DEPCAG system:

(65) y′′(t)+(κ2y2(t)−2)y′(t)−8y(t)  −κ1sin(ωt)y2(γ(t))−κ2cos(ωt)=0,

where *κ*_1_, *κ*_2_ ∈ ℝ, *γ*(*t*) = *γ*_*i*_ if *t*_*i*_ ≤ *γ*_*i*_ < *t*_*i*+1_, *i* ∈ *ℤ*, and the sequences {*t*_*i*_}_*i*∈*ℤ*_ and {*γ*_*i*_}_*i*∈*ℤ*_ satisfy the (2*π*/*ω*, *p*) condition.

We write the DEPCAG system (65) in the system form

(66) z′(t)=(0182)z(t)+(0κ1sin(ωt)z12(γ(t))) +(0κ2cos(ωt)−κ2z12(t)z2(t)),

where

(67) $$\begin{array}{c} A=\left(\begin{array}{cc} 0 & 1\\ 8 & 2 \end{array}\right),\\ f\left(t,z\left(t\right),z\left(\gamma \left(t\right)\right)\right)=\left(\begin{array}{c} 0\\ \kappa_{1}\sin\left(\omega t\right)z_{1}^{2}\left(\gamma \left(t\right)\right) \end{array}\right),\\ g\left(t,z\left(t\right),z\left(\gamma \left(t\right)\right)\right)=\left(\begin{array}{c} 0\\ \kappa_{2}\cos\left(\omega t\right)-\kappa_{2}z_{1}^{2}\left(t\right)z_{2}\left(t\right) \end{array}\right). \end{array}$$

It is easy to see that the linear homogenous system *z*′(*t*) = *Az*(*t*) does not admit any nontrivial *ω*-periodic solution; that is, the condition (*N*_*ω*_) is satisfied. Let *φ*(*t*) = (*φ*_1_(*t*), *φ*_2_(*t*)), *ψ*(*t*) = (*ψ*_1_(*t*), *ψ*_2_(*t*)) and define *𝕊* = {*z* ∈ *ℙ*_*ω*_, ||*z*|| ≤ *R*}, where *R* ∈ ℝ_+_ satisfies the condition

(68) $$\begin{array}{c} 2c_{G}R\left(\kappa_{1}+\kappa_{2}R\right)<1. \end{array}$$

Then, for *φ*, *ψ* ∈ *𝕊* we have

(69) ||g(·,φ(·),φ(γ(·)))−g(·,ψ(·),ψ(γ(·)))||≤supt∈[τ,τ+ω]|(0κ2cos(ωt)−κ2φ12(t)φ2(t))−(0κ2cos(ωt)−κ2ψ12(t)ψ2(t))|≤supt∈[τ,τ+ω]|(κ2(ψ1(t)+φ1(t))φ2(t)κ2ψ12(t))(ψ1(t)−φ1(t)ψ2(t)−φ2(t))|≤2κ2R2supt∈[τ,τ+ω]|(ψ1(t)−φ1(t)ψ2(t)−φ2(t))|=2κ2R2||φ−ψ||.

In a similar way, for *f* we have

(70) ||f(·,φ(·),φ(γ(·)))−f(·,ψ(·),ψ(γ(·)))||  ≤2κ1R||φ−ψ||.

By Theorem 14, the DEPCAG system (65) has a unique 2*π*/*ω*-periodic solution in *𝕊*.
